# Cerebrospinal fluid immune phenotyping reveals distinct immunotypes of myalgic encephalomyelitis/chronic fatigue syndrome

**DOI:** 10.1093/jimmun/vkaf087

**Published:** 2025-05-15

**Authors:** Victoria C Bastos, Kerrie A Greene, Alexandra Tabachnikova, Bornali Bhattacharjee, Per Sjögren, Bo Bertilson, Jack Reifert, Minlu Zhang, Kathy Kamath, John Shon, Jeff R Gehlhausen, Leying Guan, Michael VanElzakker, Amy Proal, Björn Bragée, Akiko Iwasaki

**Affiliations:** Department of Immunobiology, Yale School of Medicine, New Haven, CT, United States; Laboratório de Inflamação e Imunidade, Instituto de Microbiologia Paulo de Góes, Universidade Federal do Rio de Janeiro, Rio de Janeiro, Brazil; Department of Immunobiology, Yale School of Medicine, New Haven, CT, United States; Department of Immunobiology, Yale School of Medicine, New Haven, CT, United States; Department of Immunobiology, Yale School of Medicine, New Haven, CT, United States; Center for Infection and Immunity, Yale School of Medicine, New Haven, CT, United States; Department of Neurobiology, Care Sciences and Society, Karolinska Institute, Stockholm, Sweden; Bragée Clinics, Stockholm, Sweden; Department of Neurobiology, Care Sciences and Society, Karolinska Institute, Stockholm, Sweden; Bragée Clinics, Stockholm, Sweden; SerImmune Inc., Goleta, CA, United States; SerImmune Inc., Goleta, CA, United States; SerImmune Inc., Goleta, CA, United States; SerImmune Inc., Goleta, CA, United States; Department of Immunobiology, Yale School of Medicine, New Haven, CT, United States; Department of Dermatology, Yale University School of Medicine, New Haven, CT, United States; Center for Infection and Immunity, Yale School of Medicine, New Haven, CT, United States; Department of Biostatistics, Yale School of Public Health, New Haven, CT, United States; PolyBio Research Foundation, Medford, MA, United States; Division of Neurotherapeutics, Massachusetts General Hospital, Harvard Medical School, Boston, MA, United States; PolyBio Research Foundation, Medford, MA, United States; Department of Neurobiology, Care Sciences and Society, Karolinska Institute, Stockholm, Sweden; Bragée Clinics, Stockholm, Sweden; Department of Immunobiology, Yale School of Medicine, New Haven, CT, United States; Center for Infection and Immunity, Yale School of Medicine, New Haven, CT, United States; Department of Molecular, Cellular, and Developmental Biology, Yale University, New Haven, CT, United States; Howard Hughes Medical Institute, Chevy Chase, MD, United States

**Keywords:** cerebrospinal fluid, immune phenotypes, matrix metalloproteinases, ME/CFS, neuroimmunology

## Abstract

Myalgic encephalomyelitis/chronic fatigue syndrome (ME/CFS) is a complex heterogeneous multiorgan disease that can have severe impact on individuals' quality of life. Diagnosis of ME/CFS is based on symptom presentation, and a significant goal for the field is to establish meaningful subtypes. The heterogeneity in the literature suggests that individuals living with ME/CFS may suffer from overlapping but different underlying pathophysiological mechanisms. We enrolled 40 participants with ME/CFS and 41 matched healthy control subjects at the Bragée Clinic in Sweden. We assessed plasma samples from both ME/CFS cases and control groups and cerebrospinal fluid (CSF) samples from individuals with ME/CFS. We investigated dysregulated pathways and disease profiles through clinical questionnaires; multiplex analyses of cytokines, hormones, and matrix metalloproteinases; pathogen seroreactivity through peptide display bacteria libraries; and high-throughput microarray for autoantibodies. All samples used were from humans. We show altered interaction patterns between circulating biological factors in plasma of ME/CFS participants. Our analysis of CSF from individuals with ME/CFS revealed different immunotypes of disease. We found 2 patient clusters based on matrix metalloproteinases profiles. The subgroups had similar clinical presentation but distinct pathogen exposure and CSF inflammatory profiles. Our findings shed light on ME/CFS immune phenotypes and generate hypotheses for future research in disease pathogenesis and treatment development by exploring disease subgroups.

## Introduction

Myalgic encephalomyelitis/chronic fatigue syndrome (ME/CFS) is a multisystem complex disorder with major impact on the quality of life. ME/CFS comprises a broad range of manifestations including postexertional malaise, persistent fatigue, sleep dysfunction, pain, and autonomic manifestations.^1^^,^^2^ Disease onset is frequently attributed to an initial infectious insult,^3–7^ although trauma and other stressors can also be initiators. Recently, the prevalence of long COVID cases following the COVID-19 pandemic has brought further attention to postacute infectious syndromes (PAISs). While the longitudinal trajectory of ME/CFS varies greatly, most individuals see a progressive health decline with time.^8^ While some interventions may provide temporary symptomatic relief, there are no disease-modifying treatments available for ME/CFS. Studies have long sought to look for disease-specific immune signatures in peripheral blood^9–11^ and CSF.^12–14^ However, findings have often been inconsistent, and the underlying pathophysiology of ME/CFS is yet to be fully understood.

Presently, there is no well-established or widely accessible biomarker associated with ME/CFS, and patient diagnosis relies on various clinical criteria.^15^ As such, it is currently unclear whether all individuals living with ME/CFS are experiencing the same underlying disease mechanism. This may be an important confounding factor in previous research work done in the field. While studies have sought to identify subgroups of patients,^16^ mainly based on frequency and severity of symptoms,^17^^,^^18^ no clear subtypes of ME/CFS have been established based on biological parameters. Given that distinct drivers of diseases require properly targeted therapies, deeper biological phenotyping of individuals living with ME/CFS is necessary to elucidate the different subgroups within ME/CFS.

Neuroinflammation is a key target of investigation in pathogenesis of ME/CFS^19–21^ and PAISs.^22^^,^^23^ Cytokine release, activation of glial cells, and infiltration of peripheral immune cells in the CNS are known players in neuroinflammatory processes. Dysregulation of neuroinflammation can lead to sickness behavior including symptoms present in ME/CFS and PAISs.^24^^,^^25^ Additionally, matrix metalloproteinases (MMPs), enzymes involved in breakdown of extracellular matrix components, have been reported to be elevated in neurodegenerative,^26^^,^^27^ neuropsychiatric,^28^ and neurovascular^29^ disorders. MMPs are crucial in tissue remodeling but can also lead to increased blood-brain barrier permeability and impaired neuronal function.^30^^,^^31^ Here, we performed immune phenotyping of CSF and matched plasma samples looking for dysregulated pathways and distinct immunotypes within people living with ME/CFS.

## Materials and methods

### Ethics statement

This study was approved by the Swedish Ethical Review Authority (no. 2019-03510, no. 2022-06786-02).

### ME/CFS cohort and control cohort inclusion and exclusion criteria

Participants were enrolled between November 2021 and June 2022 at the Bragée Clinic in Stockholm, Sweden. Inclusion criteria for cases was age between 18 and 65 yr and previous diagnosis of ME/CFS according to the Canadian Consensus Criteria.^32^ Control subjects were sex and age matched. Control subjects were assessed for overall health and had no current usage of regular medications. Only individuals in the ME/CFS cohort underwent lumbar puncture. Exclusion criteria for all groups were pregnancy, usage of corticosteroids or any immunobiological medications, coagulopathies, and presence of contraindications for magnetic resonance imaging or lumbar puncture.

### Clinical assessments

At enrollment, an overall health assessment and a new Canadian Consensus Criteria diagnostic assessment were performed on participants by a physician. Age and sex assigned at birth were self-reported. Pain, fatigue, and brain fog (difficulty to think) were quantified by participants on a Likert scale (0–10). Memory difficulties, concentration difficulties, and sleep issues were quantified by participants on a Likert scale (0–4; 0 = none, 1 = mild, 2 = moderate, 3 = severe, 4 = unbearable). Postexertional malaise was defined as an exacerbation of some or all symptoms occurring after physical or cognitive exertion and was assessed during physician-led anamnesis. Orthostatic intolerance and postural orthostatic tachycardia syndrome (POTS) were assessed by patients’ reports and confirmed by a tilt table test. Functional status was evaluated based on standardized questionnaires EQ-5D,^33^ Quality of Life Scale,^34^ and RAND-36.^35^

#### Beighton score

Participants were assessed by a physician and given 1 point for meeting each of the following criteria: able to put palms on the floor with straight knees; left overextension of the little finger at least 90° with the palm on the table; right overextension of the little finger at least 90° with the palm on the table; left palmar flexion of wrist and bending of thumb to volar side of forearm; right palmar flexion of wrist and bending of thumb to volar side of forearm; left elbow overextension at least 10°; right elbow overextension at least 10°; left overextension of knee at least 10°; and right overextension of knee at least 10°.

#### Gait speed

Patients were asked to walk 10 m using their usual high speed that does not normally cause problems afterward. Time to walk the 10 m was measured in seconds.

#### Tilt table test

Participants were tied to a table at horizontal position with their vital signs monitored. The table was progressively tilted up to upright position while participants were monitored for symptoms, blood pressure variations, and heart rate variations. POTS was defined as the surge of symptoms (e.g. dizziness, fainting, sweating without fainting, nausea, weakness) associated with either an increase in heart rate >30 beats/min from resting heart rate or a heart rate >120 beats/min.

### Sample collection and processing

Plasma samples were derived from extraction of whole venous blood to EDTA tubes (BD Vacutainer) under standard procedures with subsequent centrifugation (2,000 *g* for 15 min, 4 °C) within 1 h from sampling and treated under refrigerated conditions during the whole process. Supernatant was transferred to cryotubes in aliquots of 250 mL and immediately frozen (−80 °C). Samples used for these analyses were not previously thawed.

CSF was collected only from ME/CFS patients through standard aseptic lumbar puncture procedure with local anesthesia and with the participant lying on the side, using sterile polypropylene tubes (Sarstedt Ag & Co) on ice. Following CSF collection, tubes were immediately centrifuged (2,000 *g*, 15 min, 4 °C) and the supernatant transferred to sterile cryotubes in 200 mL aliquots and stored in −80 °C. Samples used for these analyses were not previously thawed.

### Multiplex proteomic analysis

Both CSF and plasma samples were assessed for cytokines, hormones, and MMPs. For each sample type, only samples run simultaneously on the same assay were included for analysis. For CSF analysis, only soluble factors with at least 70% of individual values above lower levels of quantification for each marker were included. All Luminex assays were run in singleplex, and top candidates were validated by enzyme-linked immunosorbent assays.

#### Quantification of cytokines

This study used Luminex xMAP technology (Eve Technologies) for multiplexed quantification of 71 human cytokines, chemokines, and growth factors.

The multiplexing analysis was performed using the Luminex 200 system (Eve Technologies). Seventy-one markers were simultaneously measured in the samples using Eve Technologies' Human Cytokine 71-Plex Discovery Assay, which consists of 2 separate kits; one 48-plex and one 23-plex (MilliporeSigma). The assay was ran according to the manufacturer's protocol. The 48-plex consisted of sCD40L, EGF, eotaxin, FGF-2, FLT-3 ligand, fractalkine, G-CSF, GM-CSF, GROα, IFN-α2, IFN-γ, IL-1α, IL-1β, IL-1RA, IL-2, IL-3, IL-4, IL-5, IL-6, IL-7, IL-8, IL-9, IL-10, IL-12 (p40), IL-12 (p70), IL-13, IL-15, IL-17A, IL-17E/IL-25, IL-17F, IL-18, IL-22, IL-27, IP-10, MCP-1, MCP-3, M-CSF, MDC, MIG/CXCL9, MIP-1α, MIP-1β, PDGF-AA, PDGF-AB/BB, RANTES, TGFα, TNF-α, TNF-β, and VEGF-A. The 23-plex consisted of 6CKine, BCA-1, CTACK, ENA-78, eotaxin-2, eotaxin-3, I-309, IL-16, IL-20, IL-21, IL-23, IL-28A, IL-33, LIF, MCP-2, MCP-4, MIP-1δ, SCF, SDF-1α + β, TARC, TPO, TRAIL, and TSLP. Assay sensitivities of these markers range from 0.14 to 55.8 pg/mL for the 71-plex. Individual analyte sensitivity values are available in the MILLIPLEX MAP protocol (MilliporeSigma).

#### Quantification of hormones

The multiplexing analysis was performed using the Luminex 200 system. Six markers were simultaneously measured in the samples using Eve Technologies' Steroid/Thyroid Hormone 6-Plex Discovery Assay (MilliporeSigma) according to the manufacturer's protocol. The 6-plex consisted of cortisol, estradiol, progesterone, T3, T4, and testosterone. Assay sensitivities of these markers range from 0.01 to 0.24 ng/mL for the 6-plex. Individual analyte sensitivity values are available in the MILLIPLEX MAP protocol.

#### Quantification of MMPs

The multiplexing analysis was performed using the Luminex 200 system . Four markers were simultaneously measured in the samples using Eve Technologies' Human MMP Panel 2 4-Plex Custom Assay (MilliporeSigma) according to the manufacturer's protocol. The 4-plex consisted of MMP-1, MMP-2, MMP-7, and MMP-10. Assay sensitivities of these markers range from 2 to 300 pg/mL for the 4-plex. Individual analyte sensitivity values are available in the MILLIPLEX MAP protocol (HMMP2MAG-55K).

### Linear peptide profiling

#### Serum epitope repertoire analysis screening

A detailed description of the serum epitope repertoire analysis (SERA) assay has been published.^36^ For this study, plasma was incubated with a fully random 12-mer bacterial display peptide library (1 × 10^10^ diversity, 10-fold oversampled) at a 1:25 dilution in a 96-well, deep well plate format. Antibody-bound bacterial clones were selected with 50 µL Protein A/G Sera-Mag SpeedBeads (GE Healthcare Life Sciences; 17152104010350) (IgG). The selected bacterial pools were resuspended in growth media and incubated at 37  °C shaking overnight at 300 rpm to propagate the bacteria. Plasmid purification, polymerase chain reaction amplification of peptide-encoding DNA, and barcoding with well-specific indices was performed as described. Samples were normalized to a final concentration of 4 nM for each pool and run on the Illumina NextSeq 500. Every 96-well plate of samples processed for this study contained healthy control run standards to assess and evaluate assay reproducibility and possible batch effects.

The SERA assay utilizes a high-diversity, fully random 12-amino-acid peptide display library to profile antibody repertoires and identify the shared disease specific immunodominant epitopes for each organism in a single assay. All validated panels were developed as previously described.^36^ In brief, serum samples from serologically positive and serologically negative subjects were screened to identify a panel of disease specific epitopes that were generally present in ≥5% of disease sera and <1% of control subjects. To further verify epitope specificity, epitopes were evaluated in a cohort of 2,660 sera from healthy blood donors from the Serimmune database based on the expected seroprevalence. Epitope panels were subsequently validated on an independent cohort of serologically positive disease and control sera (n ≥ 30 for each). Reproducibility and repeatability studies were performed on a total of 14 unique specimens that were selected based on their antibody response levels to 8 different SERA panels. Six separate screens were performed by 3 users. Coefficient of variation (CV) values for each panel ranged from 4.6% to 11.4% (mean 7.3%) depending on the epitope panel being tested. The limit of detection (LOD) varies depending on the antibody epitope being measured and so it is not possible to determine the LOD for each panel. Panels in development were not validated on a predicate positive cohort. In these cases, panel motifs were mapped to known immunodominant epitopes/antigens reported in the literature.

For this study, all samples were screened in singlet. Each plate screened includes 2 separately run controls run in duplicate as a quality control step. The panel scores for these controls must fall within a specified range based on the assay CV for the data to pass. Additional quality controls have been established including the number of total next-generation sequencing reads for each sample and in well quality control barcodes to ensure that all steps of the assay were successful and that no cross-contamination occurred between wells.

### Autoantibody screening

For detection of antibody reactivities against known autoantigens associated with various autoimmune diseases, frozen plasma samples were shipped on dry ice to the University of Texas Southwestern Medical Center Core laboratory for microarray-based detection of both IgM and IgM antibodies against 120 autoantigens. The antigens included were: ACE2, aggrecan, albumin, alpha-fodrin, amyloid beta (1-40), amyloid beta (1-42), AQP4, BAFF, BCOADC-E2, BPI, calprotectin/S100, CD4, CD40, CENP-A, CENP-B, collagen I, collagen II, collagen III, collagen IV, collagen V, complement C1q, complement C3, complement C4, complement C5, complement C6, complement C7, complement C8, complement C9, CRP, cytochrome C, DFS70, dsDNA, EJ, factor B, factor H, factor I, factor P, fibrinogen type I-S, fibronectin, GAD65, GBM, genomic DNA, Gliadin, gp210, GP2, H/K-ATPase, histone, histone H1, histone H2A, histone H2B, histone H3, HSPG, IA-2, IF, IFN-gamma, IL-6, IL-12/NKSF, IL-17A, Jo-1, KS, KU (P70/P80), La/SS-B, laminin, LC1, LKM 1, LPS, lysozyme, M2, MBP, MDA5, Mi-2, mitochondrion, MPO, myosin, Nrp1, nucleolin, nucleosome, Nup 62, NXP2, OGDC-E2, P0, P1, P2, PCNA, PDC-E2, PL-7, PL-12, PM/Scl-75, PM/Scl 100, PR3, proteoglycan, prothrombin, Ro/SS-A (52 kDa), Ro/SS-A (60 kDa), SAE1/SAE2, Scl-70, SLA/LP, Sm, Sm/RNP, SmD, SmD1, SmD2, SmD3, SP100, SRP54, ssDNA, tau, thyroglobulin, TIF1 gamma, TLR4, TNF-alpha, TPO, tTG, U1-snRNP 68/70 kDa, U1-snRNP A, U1-snRNP C, U-snRNP B/B', vimentin, vitronectin, and β2-glycoprotein 1. Plasma samples were pretreated with DNAse I and subsequently diluted as described previously.^37^ The microarray slides were scanned using GenePix 4200A instrument (Molecular Devices). The GenePix Pro 7 Microarray Acquisition and Analysis Software was used to acquire and analyze array images. For each plasma sample, net signal intensity and signal-to-noise ratio (SNR) were generated for each antigen. Antigens that had >3 SNR values for <15% of the samples were excluded from analyses. Antibody scores were calculated using the formula log_2_(net signal intensity*SNR + 1) as a measurement of binding capacity. All assays were run in singleplex, and top candidates were validated by enzyme-linked immunosorbent assays. Cases and controls were run together to avoid batch effects.

### Statistical analysis

For data comparing healthy individuals and ME/CFS individuals, comparisons were made using 2-sided Wilcoxon rank sum testing, with correction for multiple comparisons made using Bonferroni post hoc testing. For dot plots, the central lines indicate the group median and the top and bottom lines indicate the 75th and 25th percentiles, respectively. In these comparisons, accounting for age, sex assigned at birth, and body mass index (BMI) between groups was performed through analysis of covariance. Differences in autoantibody levels were assessed using Mann-Whitney *U* tests with corrections for multiple testing using the Benjamini-Hochberg method. Correlation analysis was performed through Pearson correlation. All correlations shown were filtered for significance (unadjusted *P* ≤ 0.05) using hypothesis testing of Pearson correlation. Unadjusted *P* values are commonly used in a correlation analysis and were solely used for correlation matrix filtering. The heatmap was produced using unsupervised hierarchical clustering. Generalized linear modeling was performed linear regression with outputs of model coefficients for each variable and associated *P* value. All statistical testing was performed using JMP Pro 17.0.0 (StataCorp), Prism 10.4.1 (GraphPad Software) or R 4.4.2 (R Foundation for Statistical Computing. The *P*-value adjustment method for multiple comparison choice was influenced by optimizing conservativeness and software implementation ability. Any additional statistical methods are annotated in figure legends.

## Results

### Study design, demographics, and clinical features

**Figure 1.**
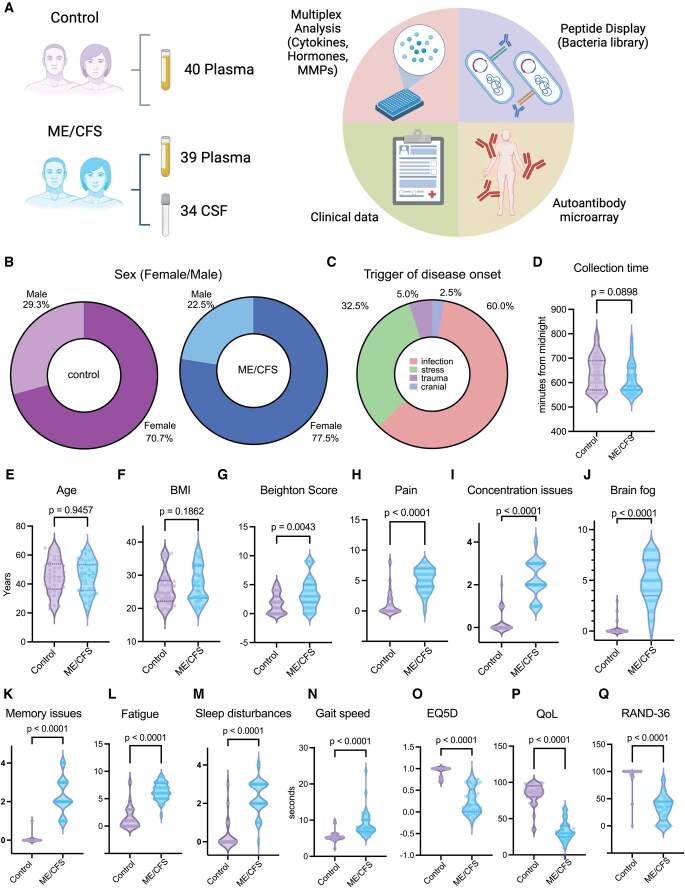
Demographics and clinical parameters of individuals with ME/CFS. (A) Schematic of study design. Numbers indicate samples collected. Diagram created using BioRender. (B) Distribution of sex assigned at birth for control subjects (left, purple) and participants with ME/CFS (right, blue). n = 41 (control subjects) and n = 39 (ME/CFS participants). (C) Reported triggers of disease onset within the ME/CFS participants. (D–F) (D) Sample collection time in minutes from midnight, (E) age in years, and (F) BMI for control subjects (left, purple) and participants with ME/CFS (right, blue). (G) Assessed Beighton scores for control subjects (left, purple) and participants with ME/CFS (right, blue). (H–M) Reported levels of (H) pain, (I) concentration issues, (J) brain fog, (K) memory issues, (L) fatigue, and (M) sleep disturbances from control subjects (left, purple) and participants with ME/CFS (right, blue). (N) Measured gait speed reported in total seconds taken to walk 10 m for control subjects (left, purple) and participants with ME/CFS (right, blue). (O–Q) Assessed scores of (O) EQ-5D, (P) Quality of Life Score (QoL), and (Q) RAND-36 for control subjects (left, purple) and participants with ME/CFS (right, blue). Each dot represents 1 participant. The central lines indicate the group median and the top and bottom lines indicate the 75th and 25th percentiles, respectively. Significance for differences between groups was assessed using 2-sided Wilcoxon rank sum test.

The study enrolled 81 participants (40 ME/CFS patients, 41 healthy control subjects) at the Bragée Clinic. Samples were successfully collected from 79 participants (39 ME/CFS participants, 40 healthy control subjects) at rest between November of 2021 and June of 2022. CSF samples were collected from 34 participants in the ME/CFS group through lumbar puncture. The study comprised clinical questionnaires, multiplexed analysis of cytokines, measurements of hormones and MMPs, antibodies against pathogens, and autoantibodies (Fig. 1A). Both groups contained higher number of female participants (70.7% in healthy control subjects and 77.5% in ME/CFS participants) (Fig. 1B) than males. Among the self-reported triggers of disease onset, infection (60%) and stress (32.5%) were the most common (Fig. 1C). Sample collection time of day, age, and BMI did not differ significantly between groups (Fig. 1D–F). Upon clinical assessment, ME/CFS cases displayed significantly higher Beighton score total values (Fig. 1G); higher self-reported levels of pain, concentration issues, brain fog, memory issues, fatigue, and sleep disturbances (Fig. 1H–M); and slower gait speed measurements (measured in seconds taken to walk 10 m) (Fig. 1N) compared with control subjects. Additionally, 3 commonly used standardized quality-of-life questionnaires were administered to the cohort: EQ-5D,^33^ Quality of Life Scale,^34^ and RAND-36.^35^ ME/CFS participants scored lower than control subjects across all 3 performed surveys (Fig. 1O–Q).

### ME/CFS participants displayed similar prior exposure to selected exogenous pathogens as control subjects

Exposure to various pathogens has long been hypothesized to contribute to the development of ME/CFS. To interrogate past exposure to parasitic, viral, and tick-borne bacterial pathogens previously described to be prevalent in populations of ME/CFS patients^38–40^ or associated with PAISs,^41^ we employed SERA. SERA is a commercially available bacterial display library which encompasses linear epitopes representing common pathogens. It leverages a database of thousands of infected vs. seronegative control subjects to determine past exposure to a pathogen using antibody repertoires in serum or plasma.^36^ For most pathogens, control subjects and ME/CFS participants did not differ in frequency of past exposure (Fig. S1). Significantly more study participants with ME/CFS had a prior exposure to Dengue virus than control subjects (*P* = 0.0436), possibly due to travel history. However, this represented a very small number of participants (n = 2 in the ME/CFS group and n = 0 in the control group).

### Evaluation of autoantibodies in patients with ME/CFS

Multiple studies have demonstrated the heightened presence of autoantibodies in ME/CFS, often in association with an infectious onset.^40^^,^^41^ Hence, next we screened for autoantibody reactivities in the plasma using high-throughput autoantigen microarrays. Presence of both IgM and IgG isotypes were evaluated. To reduce false positive detection, autoantibodies with SNR >3 in more than 15% of the samples were excluded from further analysis. For the plasma IgM analyses, 2 antigens had to be excluded for exceeding the SNR cutoff. Out of the 118 antigens, significantly higher IgM autoantibody reactivities were detected in healthy individuals compared with participants with ME/CFS for 57 antigens. However, the log_2_ fold changes were very modest (−0.049 to 0.47) (Fig. S2A). Factor P was the autoantigen with the highest differential IgM reactivity, with a log_2_(fold change) of 0.47 (*P* = 0.049) (Fig. S2B). Additionally, anti-factor P IgM was significantly lower in participants with ME/CFS when compared with healthy control subjects (*P* = 0.0232). (Fig. S2C).

### ME/CFS participants displayed distinct correlation network signatures of plasma soluble factors

**Figure 2.**
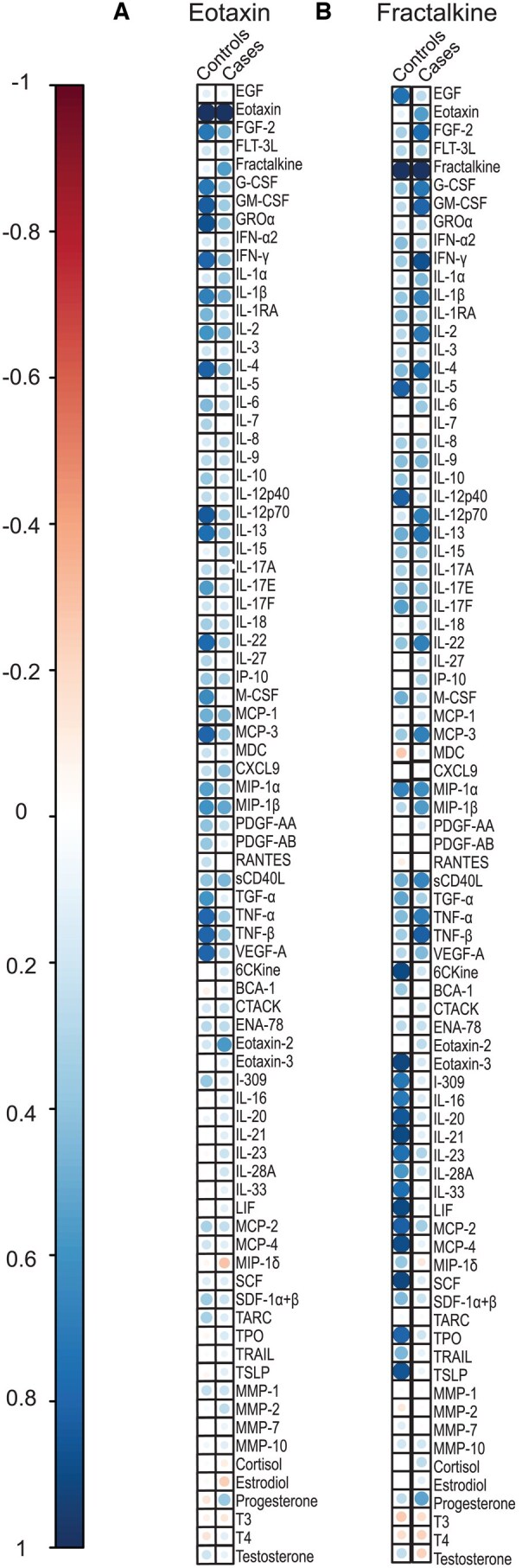
Correlation of soluble factors in ME/CFS. (A, B) Correlations between eotaxin (A) and fractalkine (B) and 91 soluble factors including cytokines, hormones, and MMPs from plasma samples for control subjects and participants with ME/CFS (cases). Only significant correlations (*P* < 0.05) are represented as colored dots. Empty (white) squares represent a lack of statistically significant correlation between 2 markers within the respective group. Pearson’s correlation coefficients from comparisons of soluble factors’ measurements within the same participants are visualized by color intensity.

We assessed plasma samples from individuals with and without ME/CFS for circulating levels of immune mediators, hormones, and MMPs. Except for TRAIL (Fig. S3A), no significant differences in total plasma levels for any of these markers were identified between participants with ME/CFS and control subjects. To further elucidate possible differences in the plasma factor correlations within the groups, we next analyzed the network of interactions between these individual markers. We performed separate correlation matrices on all analyzed factors for control subjects and ME/CFS (Fig. S3B). Interestingly, the correlation matrix showed an overall trend for stronger positive correlations between cytokines within the ME/CFS cohort by applying a significance threshold (*P* < 0.05) to the differential correlation matrices. We found a set of correlating networks that were distinct from control subjects (Fig. 2). Correlations associated with fractalkine were present in control subjects but absent in the ME/CFS group (Fig. S4). Additionally, correlations associated with eotaxin, also known as CCL11, were present in control subjects but absent in the ME/CFS group.

### ME/CFS CSF revealed 2 subsets with distinct immunophenotypes

**Figure 3.**
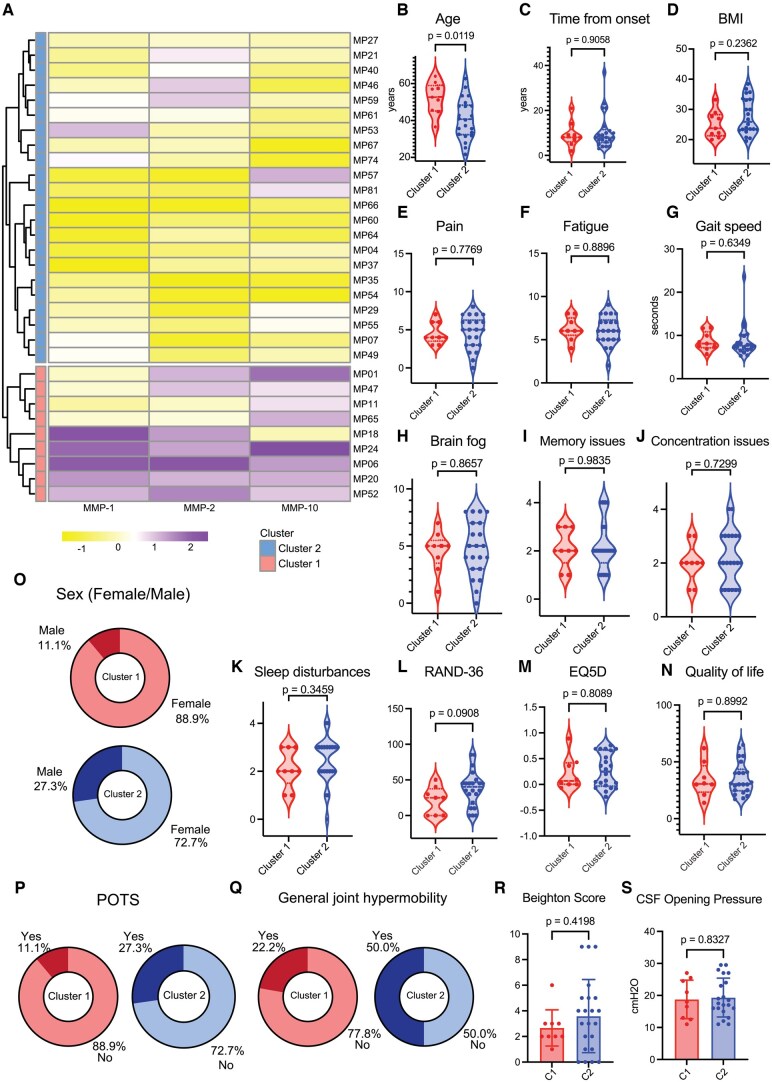
CSF MMP signature differentiates 2 subgroups of ME/CFS patients. (A) Unsupervised hierarchical clustering of participants with ME/CFS (n = 31) based on MMP-1, MMP-2, and MMP-10 measurements from CSF samples. Individuals are arranged across rows, with each colored unit indicating normalized CSF MMP quantification. Rows are further annotated by cluster 1 (n = 9, bottom, red) and cluster 2 (n = 22, top, blue). (B–D) Distribution of (B) age in years, (C) reported time from disease onset (years) at time of sample collection, and (D) BMI for cluster 1 (left, red) and cluster 2 (right, blue). (E, F) Reported levels of (E) pain and (F) fatigue from cluster 1 (left, red) and cluster 2 (right, blue). (G) Measured gait speed reported in total seconds taken to walk 10 m for cluster 1 (left, red) and cluster 2 (right, blue). (H–K) Reported levels of (H) brain fog, (I) memory issues, (J) concentration issues, and (K) sleep disturbances for cluster 1 (left, red) and cluster 2 (right, blue). (L–N) Assessed scores of (L) EQ-5D, (M) Quality of Life Scale, and (N) RAND-36 for cluster 1 (left, red) and cluster 2 (right, blue). (O) Distribution of sex assigned at birth for cluster 1 (left, red) and cluster 2 (right, blue, nonsignificant). (P, Q) Prevalence of POTS (P) and general joint hypermobility (Q) for cluster 1 (left panel, red) and cluster 2 (right panel, blue, nonsignificant). (R, S) Assessed Beighton scores (R) and CSF opening pressures (S) for cluster 1 (left, red) and cluster 2 (right, blue). For dot plots, the central lines indicate the group median and the top and bottom lines indicate the 75th and 25th percentiles, respectively. Significance for differences between groups was assessed using 2-sided Wilcoxon rank sum test. Each dot represents 1 participant. CMV, cytomegalovirus.

We next aimed to assess whether there were distinct disease signatures within our cohort that were possibly masked by similar clinical presentations. For that, we assessed the CSF samples within individuals with ME/CFS to investigate local parameters in the central nervous system milieu. Given their role in neuroinflammation and in blood-brain barrier permeability, we selected the panel of metalloproteinases to perform unsupervised hierarchical clustering of participants (Fig. 3A). This revealed a subset of ME/CFS patients containing higher levels of MMP-1, MMP-2, and MMP-10 in CSF (cluster 1), compared with the rest (cluster 2) (Fig. S5A–C). We investigated whether these clusters were correlated with a difference in demographic and disease presentation. Though patients in cluster 1 were older (Fig. 3B), they showed no difference in reported time from disease onset or BMI (Fig. 3C, D) from patients in cluster 2. Reported levels of pain and fatigue (Fig. 3E, F) were comparable between clusters. We found no differences in gait speed or self-reported levels of brain fog, memory issues, concentration issues, or sleep disturbances (Fig. 3G–K). Both groups presented with comparable scores across all assessed methods of quality-of-life measurements (Fig. 3L, M). While cluster 1 had 11.1% of male participants and cluster 2 had 27.3% (Fig. 3O), this difference was not significant possibly due to the small sample size. A similar pattern was found regarding POTS (11.1% in cluster 1 and 27.3% in cluster 2) (Fig. 3P) and general joint hypermobility (22.2% in cluster 1 and 50% in cluster 2) (Fig. 3Q). Accordingly, total individual Beighton score values did not differ between clusters (Fig. 3R), nor did CSF opening pressure (Fig. 3S). Finally, infectious onset was the primary reported trigger of disease in both clusters.

### Different immunotypes of ME/CFS presented with distinct pathogen reactivity and CSF inflammatory signatures

**Figure 4.**
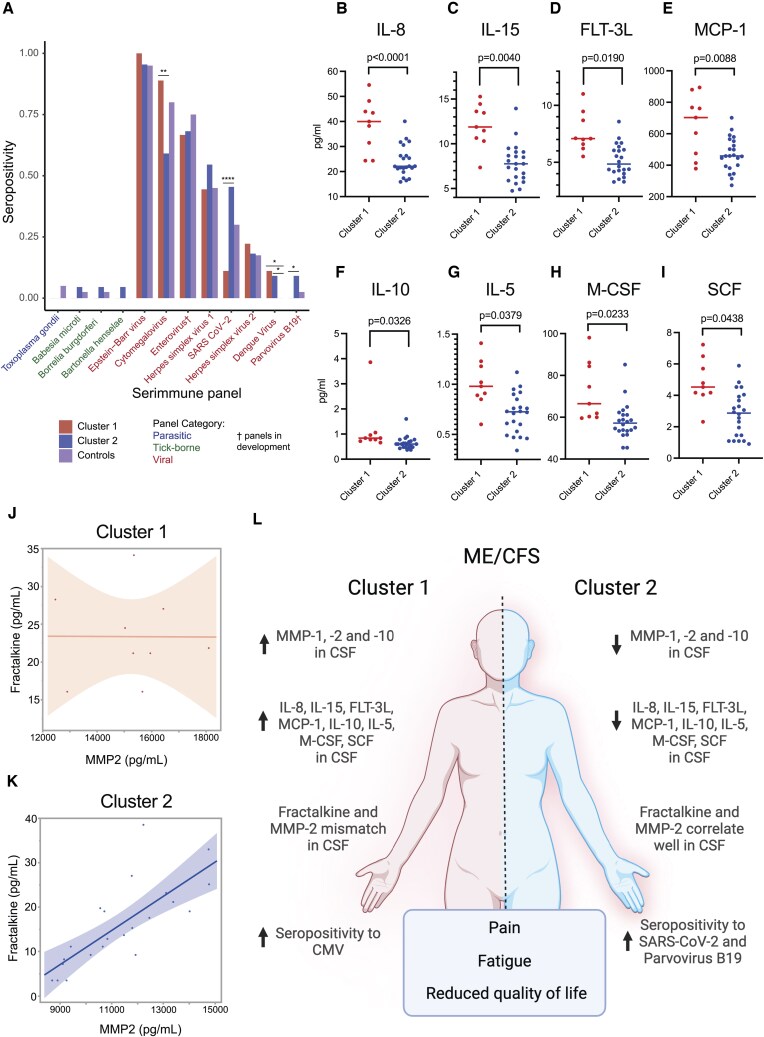
Pathogen reactivity and quantification of cytokines between subgroups in ME/CFS patients. (A) Proportion of each ME/CFS cluster and control subjects (ME/CFS cluster A: n = 9; ME/CFS cluster B: n = 22; control group: n = 40) seropositive for each of 12 common pathogen panels as determined by SERA, grouped by pathogen type. Statistical significance was determined by Fisher’s exact test corrected with false discovery rate (Benjamini-Hochberg). *P ≤ 0.05; **P ≤ 0.01; ****P ≤ 0.0001. (B–I) Quantification of cytokines (B) IL-8, (C) IL-15, (D) FLT-3L, (E) MCP-1, (F) IL-10, (G) IL-5, (H) M-CSF, and (I) SCF in CSF samples from cluster 1 (left, red) and cluster 2 (right, blue). Multiple comparison adjustment was performed using the false discovery rate method. Significance was calculated by 2-sample *t* testing accounting for variations in age, sex assigned at birth, and BMI between clusters through analysis of covariance. (J, K) Spearman’s correlation plots for measurements of fractalkine and MMP-2 in CSF samples from (J) cluster 1 (red) and (I) cluster 2 (Spearman’s ρ = 0.8158, *P* < 0.0001). (L) Schematic overview of main findings between clusters. Created with BioRender.

We assessed differences between the 2 clusters in seroprevalence for pathogen panel through the SERA platform analysis (Fig. 4A). We found that cluster 1 showed a significantly higher percentage of patients who are seropositive for cytomegalovirus. On the other hand, cluster 2 had a higher percentage of patients seropositive for SARS-CoV-2 and parvovirus B19. Interestingly, viral infections have been shown to influence MMP expression and activity.^42^^,^^43^ While patients in both clusters were indistinguishable by clinical presentation, these data showed that there might be variations in neuroinflammation and previous pathogen exposure relating to the distinct subcohorts of ME/CFS patients.

We next expanded our analysis to investigate different cytokine signatures in CSF samples between clusters and found the top 8 cytokines separating the 2 groups. These 8 cytokines were selected as distinguished markers based on a nominal false discovery cate cutoff at 0.05. Notably, all 8 cytokines (IL-8, IL-15, FLT-3L, MCP-1, M-CSF, SCF, IL-10, and IL-5) were increased in CSF samples from cluster 1 patients when compared with cluster 2 after adjustment for age, sex assigned at birth, and BMI (Fig. 4B–I). Though there were no differences in total levels of soluble fractalkine between clusters, IL-15 is a known regulator of its receptor CX3CR1^44^^,^^45^ and was detectable in higher levels in cluster 1 samples (Fig. 4C). Given the previously described association between MMP-2 and cleavage of fractalkine to its soluble form,^46^ we investigated whether this correlation was present in our cohort. While cluster 2 displayed a clear positive correlation between these markers in CSF, the same pattern was lacking in samples from cluster 1 (Fig. 4J, K). The small sample size of the clusters impaired the ability to perform correlation matrix analysis as was done for the plasma samples. The differences in cytokines seen locally in the CSF samples were not reflected in the plasma values for cytokines, consistent with prior studies.^21^ Thus, we observed that cluster 1 presented with heightened levels of both MMPs and proinflammatory cytokines within the CSF but not in the plasma. Within the plasma, we identify 1 proinflammatory cytokine, IL-7, significantly elevated in cluster 1 compared with cluster 2 and healthy control subjects (Fig. S5D). These data suggest the presence of different immunotypes of ME/CFS presenting with similar clinical presentation but possible distinct underlying mechanisms.

## Discussion

Understanding the pathophysiology of ME/CFS is paramount in subtyping and improving treatment options for this disease. Here, we showcase the investigation of ME/CFS through clinical questionnaires; plasma and CSF cytokines, hormones, and MMPs; antibody responses to exogenous pathogens; and autoantibodies. Our comprehensive immune phenotyping of CSF and plasma samples from people living with ME/CFS revealed key potential clues to further elucidate subgroups and dysregulated pathways that might drive disease.

Upon examination of clinical symptoms, we found that individuals with ME/CFS reported higher pain and fatigue levels, while also demonstrating slower gait speeds than healthy control subjects. Additionally, individuals with ME/CFS scored lower on all performed standardized quality-of-life assessments. Within our cohort, the self-reported triggers of disease onset were dominated by infection (60%) and stress (32.5%). However, when evaluating previous pathogen exposure, we found that ME/CFS and healthy individuals did not differ in exposure to most assessed pathogens. Importantly, this finding does not rule out the possibility that pathogen activity after exposure could differ between cases and controls. For example, genetic material or antigens created by infecting pathogens might persist in ME/CFS tissue or host cells. These persistent antigens could dysregulate the immune response or drive a range of other physiological abnormalities underlying ME/CFS symptoms. Indeed, immune profiling performed in a recent National Institutes of Health study suggested chronic antigenic stimulation in ME/CFS, associated with increase in naive and decrease in switched memory B cells.^47^ Other teams have identified persistent enterovirus RNA or antigen in ME/CFS^48^ gut,^49^ muscle,^50^ or brain tissue.^51^ Thus, tissue biopsy studies or the use of tetramer assays capable of identifying persistent antigen and/or immune responses indicative of antigen stimulation in ME/CFS are warranted in future research.

Many studies have investigated ME/CFS from the lens of autoimmunity,^52^^,^^53^ which may result from molecular mimicry between host and pathogen antigens.^54^ We found no significant differences between IgG autoantibodies between the plasma and CSF of ME/CFS and healthy individuals. However, we observed generally lower IgM autoantibody reactivities in ME/CFS when compared with healthy control subjects. We show here that participants with ME/CFS present with lower anti-factor P IgM levels when compared with healthy control subjects, although with low log_2_ fold change. Whether this subtle difference in the autoantibodies would result in any physiological impact is unknown. Others have reported a number of autoantibody reactivities in people with ME/CFS.^15^^,^^55^ Interestingly, treatment of patients with ME/CFS with an anti-CD20 antibody, rituximab, a B cell depletion agent and treatment option for the autoimmune disease multiple sclerosis, has failed a phase 3 clinical trial despite positive efficacy in phase 2 trials.^56^ Our analysis only included autoantigens that are frequently found in other autoimmune diseases but was not a comprehensive analysis of autoantibodies. Future studies are needed to determine whether autoantibodies that underpin disease pathogenesis are present in ME/CFS.

We found mostly increased positive correlations in circulating plasma factors present in ME/CFS when compared with healthy control subjects. The alteration in correlation signatures might be associated with a variation in induction patterns of these cytokines in individuals with ME/CFS. Plasma fractalkine and eotaxin, also known as CCL11, showed a distinct pattern of positive correlations present in control subjects but absent in participants with ME/CFS. Fractalkine can be expressed as a transmembrane protein in cells such as mature neurons and endothelial cells. In the CNS, fractalkine's interaction with its receptor (CX3CR1) can mediate neuron-microglia interactions and has been shown to affect demyelination in multiple sclerosis animal models.^57–59^ Fractalkine contains a mucin-like stalk and is bound on the cell surface. When cleaved into soluble chemokine by a variety of proteases,^60^ including MMP-2,^46^ it is a chemoattractant to T cells and monocytes. A disruption in the pathway of interactions between fractalkine and CX3CR1 in glia and circulating cells in ME/CFS patients could associate with altered local central nervous system inflammatory responses and, possibly, drive symptomatology. Notably, altered expression of CX3CR1 has been demonstrated in individuals living with ME/CFS.^11^ Altered levels of eotaxin when compared with other cytokines might be an important clue as well. Referred to as the “aging factor,”^61^ eotaxin has been associated with neurodegeneration^62^ and impaired memory.^63^ Eotaxin can transverse the blood-brain barrier^64^ and is sufficient to replicate postinfectious neuroinflammatory changes in mice.^22^ It is common practice in the clinical setting to guide diagnosis and disease stratification by the proportion between analytes.^65–67^ In the ME/CFS field, whether a ratio between different analytes could work as a biomarker of disease should be investigated in the future.

Analysis of CSF MMPs reveal 2 distinct subsets of ME/CFS within our cohort. We applied different strategies to test whether these groups could be driven by confounding factors. The groups did not differ in time from disease onset and did not differ in clinical presentation. Though participants with the consistent significantly higher signature of MMP-1, MMP-2, and MMP-10 (cluster 1) were older, the difference in MMP levels was still significant after adjustment for age, sex assigned at birth, and BMI. Additionally, cluster 1 also presented with higher levels of 8 measured cytokines (IL-8, IL-15, FLT-3L, MCP-1, M-CSF, SCF, IL-10, and IL-5). This might correlate with a disease phenotype more associated with local alterations in central nervous system inflammatory milieu. Moreover, previous studies have documented craniocervical instability in patients with ME/CFS.^68^ We postulate that instability and obstructions at the craniocervical junction may contribute to impaired movement and production of CSF, in turn altering MMP or cytokine levels in either cluster. While total fractalkine levels were comparable between clusters, elevated levels of IL-15 might correlate with an altered expression of CX3CR1. In addition, the 2 clusters displayed a distinct pattern of correlation between soluble fractalkine and MMP-2. This might correlate with different patterns of membrane-bound fractalkine cleavage between the subgroups. Further work is warranted to investigate whether, for example, cluster 1 immunotype of ME/CFS could be amenable to targeting by immunomodulatory agents that can access the CNS.^69–71^

Many groups have investigated infection as a trigger of ME/CFS.^15^^,^^47^ We found that cluster 1 showed increased seropositivity to cytomegalovirus relative to cluster 2, whereas cluster 2 showed increased seropositivity to SARS-CoV-2 and parvovirus B19 relative to cluster 1. Each other of these viral pathogens have been previously reported to trigger ME/CFS, also known as postinfectious ME/CFS.^38–40^^,^^72^ Interestingly, parvovirus B19–induced ME/CFS has been linked to joint arthralgias.^38^ In cluster 2, half of the patients had a diagnosis of general joint hypermobility. In total, these results may suggest a link between pathogen exposure and symptom presentation within ME/CFS. Still, it is noticeable that there was no clear significant difference in clinical parameters between the subgroups. In addition, seropositivity in no way indicates that these pathogens were responsible triggers of disease. Future work should consider the importance of defining subgroups of patients to elucidate possible distinct drivers of disease.

This study for the first time found 2 distinct immunotypes of ME/CFS patients based on CSF marker analysis. Even with similar clinical phenotype, diving deeper into subsets of ME/CFS based on biological markers is indispensable for identifying targeted therapies based on the underlying root causes. We hope that further differentiating ME/CFS pushes the field into subclassifying this disease. This path may lead to a better understanding of this heterogeneous disease and subsequent development of individualized efficient treatment options for individuals suffering with ME/CFS.

### Limitations of study

There are several limitations of this current study. There is a lack of CSF samples from healthy individuals as controls due to the invasive nature of the lumbar puncture procedure. Given the clear immunotypes observed in the CSF, our study underscores the true value of the ability to obtain CSF from individuals with ME/CFS. In future studies, it would be important to continue to search deeper into tissue samples,^73^ as has been done for other PAISs.^74^^,^^75^ Additionally, our analyses were restricted by the limited number of study participants. For instance, a larger sample size would allow for the assessment of correlation patterns within the subgroups of patients. Future studies should seek to continue investigating subcohorts of ME/CFS to further strengthen our understanding of possible immunotypes within this disease. Expanding our CSF MMP analysis outside of MMP-1, MMP-2, and MMP-10 may aid in the subtyping of ME/CFS. Specifically, investigating MMP-9, which plays a key role in neuroinflammation,^76^ would have added value to this study. ME/CFS studies are currently marked by the limitations of relying on clinical characteristics based on patients’ reports and anamnestic assessments. In this study, symptom description was limited. Application of additional standardized symptom and functioning questionnaires would have allowed for a more extensive evaluation of the cohort. Furthermore, future work into identifying standardized measurable biomarkers will be of importance for knowledge advancement in the field. With these limitations, we were still able to provide new insights and potentially begin to elucidate the pathophysiology of ME/CFS disease.

## Supplementary Material

vkaf087_Supplementary_Data

## Data Availability

The data underlining this article will be shared on reasonable request to the corresponding author.
