# Supplementary material for: Cerebrospinal fluid immune phenotyping reveals distinct immunotypes of Myalgic Encephalomyelitis/Chronic Fatigue Syndrome
Source: J Immunol. Author manuscript; Available in PMC 2025 Aug 1. (PMC12311384; doi:10.1093/jimmun/vkaf087)
Supplement: 1 [file NIHMS2072857-supplement-1.pdf]

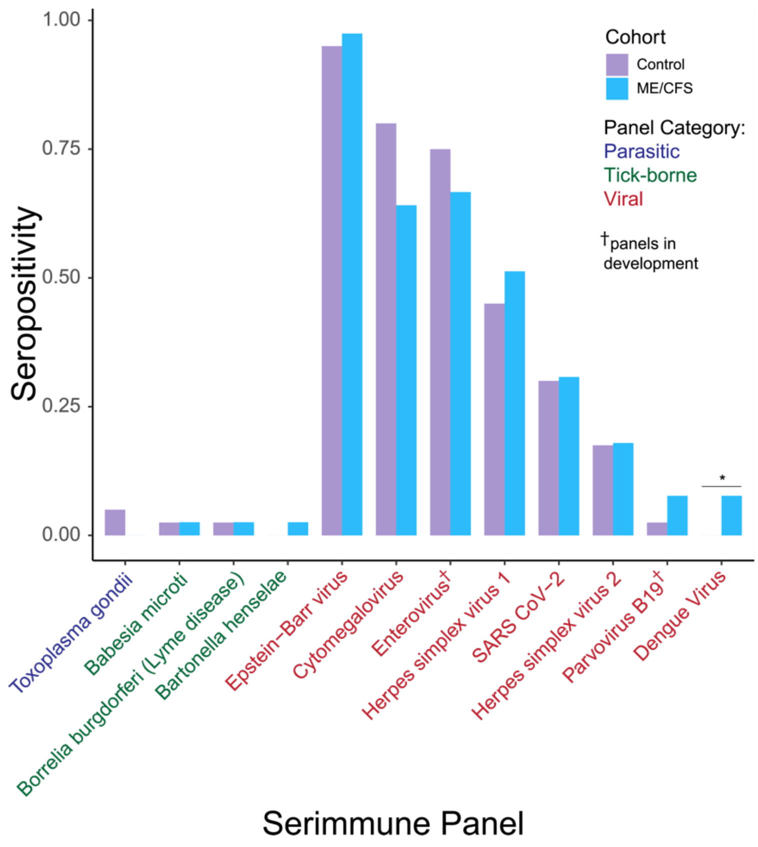

Supplemental Figure 1. Proportion of each group (ME/CFS: n = 39, control: n = 40) seropositive for each of 12 common pathogen panels as determined by SERA, grouped by pathogen-type. Statistical significance determined by Fisher's exact test corrected with FDR (Benjamini-Hochberg). (*) $p \leq .05$.

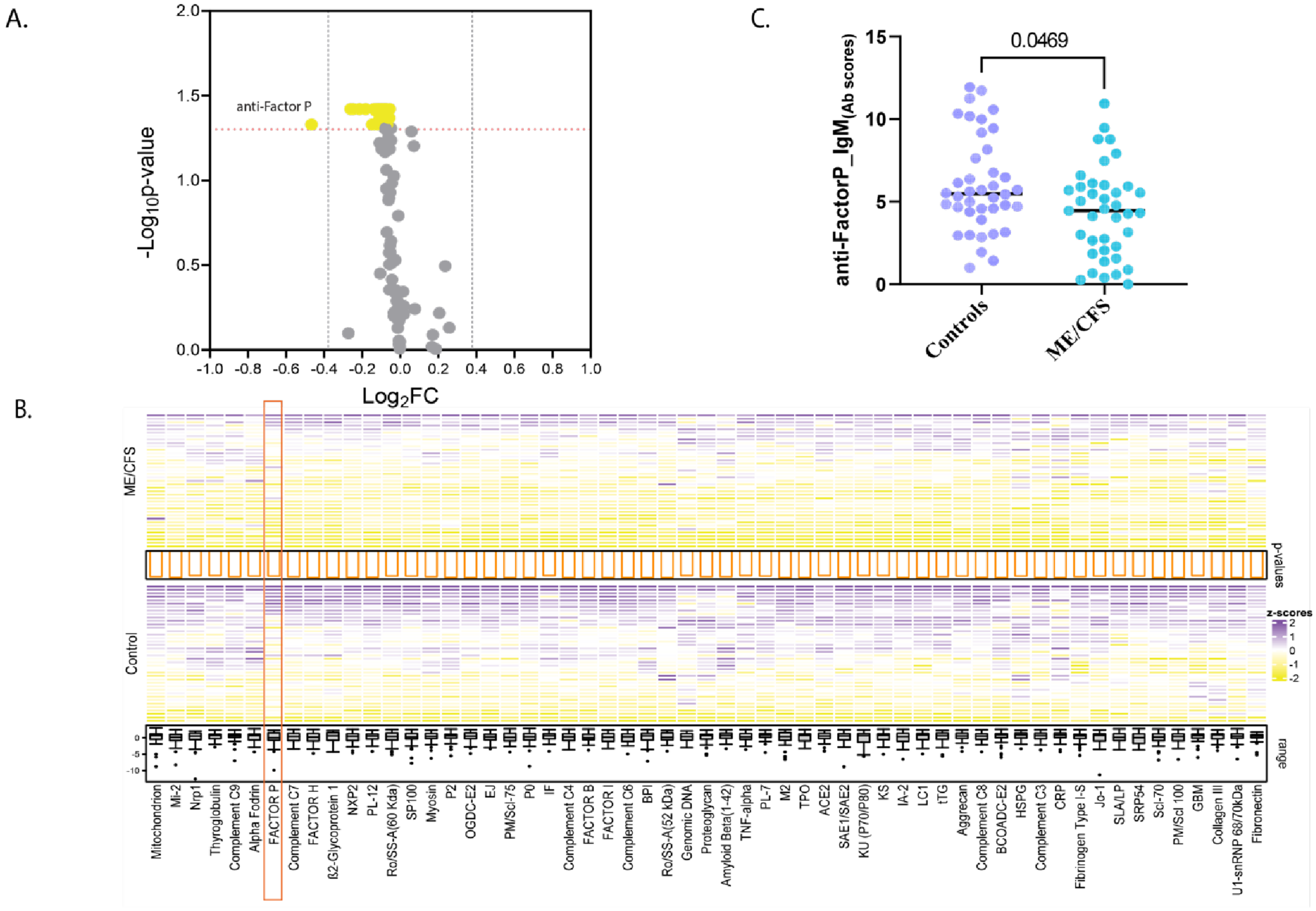

Supplementary Figure 2. A, Volcano plot showing the differential log_2(FC) IgM antibody scores for 118 known autoantigens among cases and controls. The red dotted line indicates log_{10}(p) of 0.05. Each dot represents an autoantibody and those in yellow indicate the ones that differ significantly as determined by Mann-Whitney U tests and corrected for multiple testing correction with FDR (Benjamini-Hochberg). **B**, Heatmap of z-scores showing the 57 anti-IgM autoantibodies which differ significantly between cases and controls. Each row in the first and third panel represents a participant from the ME/CFS and control cohorts respectively. The orange box highlights anti Factor-P IgM. The box plots indicate the range of z scores for each autoantibody. **C,** Comparison of anti-Factor-P IgM autoreactivities in the plasma among different cohorts. Statistical significance determined by Mann-Whitney U tests and corrected for multiple testing correction with FDR (Benjamini-Hochberg).

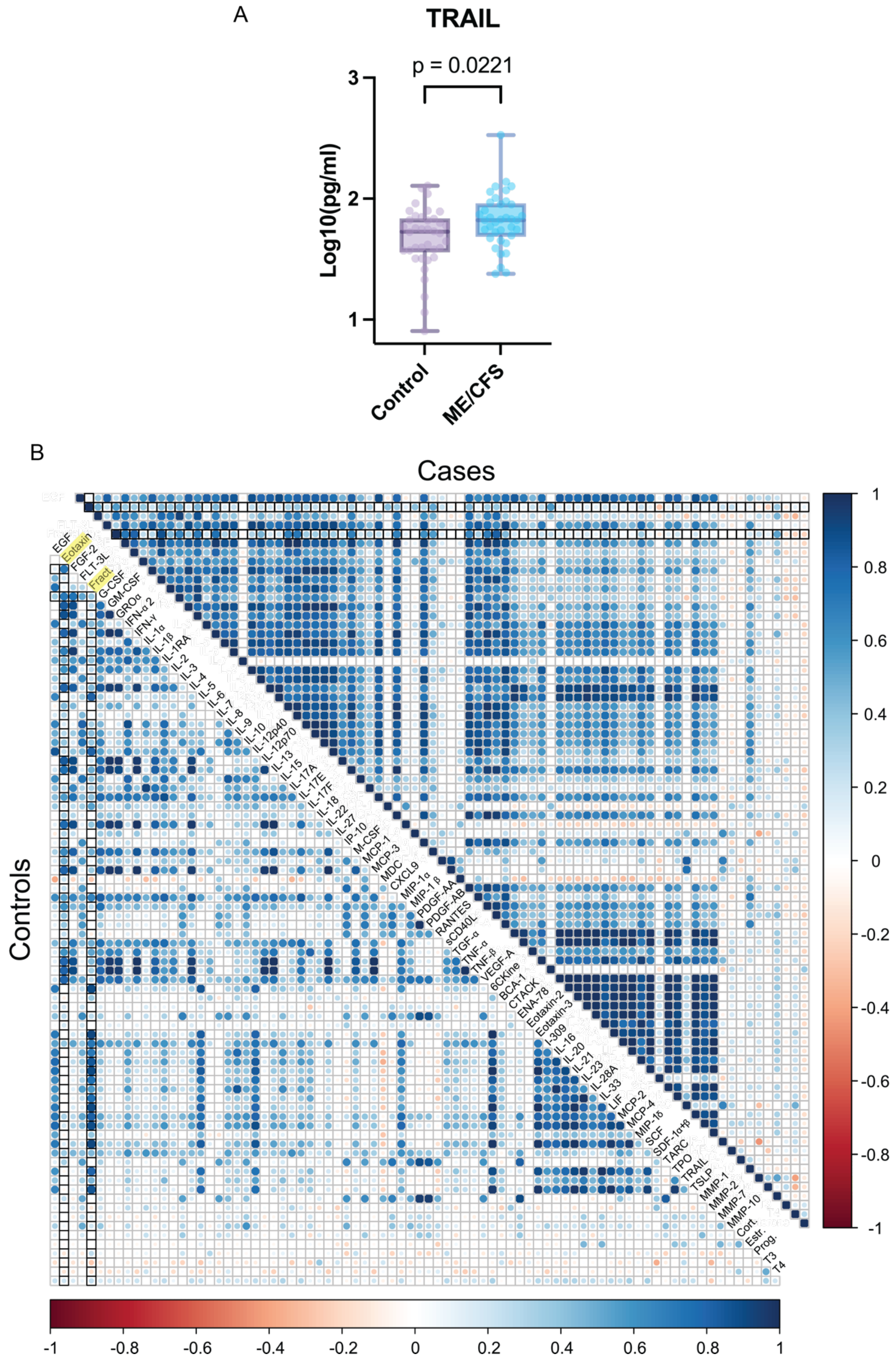
A
TRAIL
p = 0.0221
Log10(pg/ml)
3
2
1
Control
ME/CFS
B
Cases
Controls
EGF
Eotaxin
FGF-2
FLT-3L
Fract.
G-CSF
GM-CSF
GROα
IFN-α2
IFN-γ
IL-1α
IL-1β
IL-1RA
IL-2
IL-3
IL-4
IL-5
IL-6
IL-7
IL-8
IL-9
IL-10
IL-12p40
IL-12p70
IL-13
IL-15
IL-17A
IL-17E
IL-17F
IL-18
IL-22
IL-27
IP-10
M-CSF
MCP-1
MCP-3
MDC
CXCL9
MIP-1α
MIP-1β
PDGF-AB
RANTES
sCD40L
TGF-α
TNF-α
TNF-β
VEGF-A
6CKine
BCA-1
CTACK
ENA-78
Eotaxin-2
Eotaxin-3
I-309
IL-16
IL-20
IL-21
IL-23
IL-28A
IL-33
LIF
MCP-2
MCP-4
MIP-1δ
SCF
SDF-1α+β
TARC
TPO
TRAIL
TSLP
MMP-1
MMP-2
MMP-7
MMP-10
Cort.
Estr.
Prog.
T3
T4
1
0.8
0.6
0.4
0.2
0
-0.2
-0.4
-0.6
-0.8
-1

Supplementary Figure 3. A, Quantification of TRAIL in the plasma of individuals with ME/CFS and healthy controls. Multiple comparison adjustment was performed using the False Discovery Rate method. Significance was calculated by two sample t-testing accounting for variations in age, sex assigned at birth, and BMI between clusters through analysis of covariance (ANCOVA). **B,** Correlation matrices of 91 soluble factors including cytokines, hormones, and matrix metalloproteinases (MMPs) from plasma samples for controls (bottom triangle) and participants with ME/CFS (top triangle). Only significant correlations ($p<0.05$) are represented as dots. Empty squares represent a lack of statistically significant correlation between two markers within the respective group. Pearson's correlation coefficients from comparisons of soluble factors' measurements within the same participants are visualized by color intensity. Legend: Fract. = Fractalkine, Cort. = Cortisol, Estr. = Estrogen, Prog. = Progesterone.

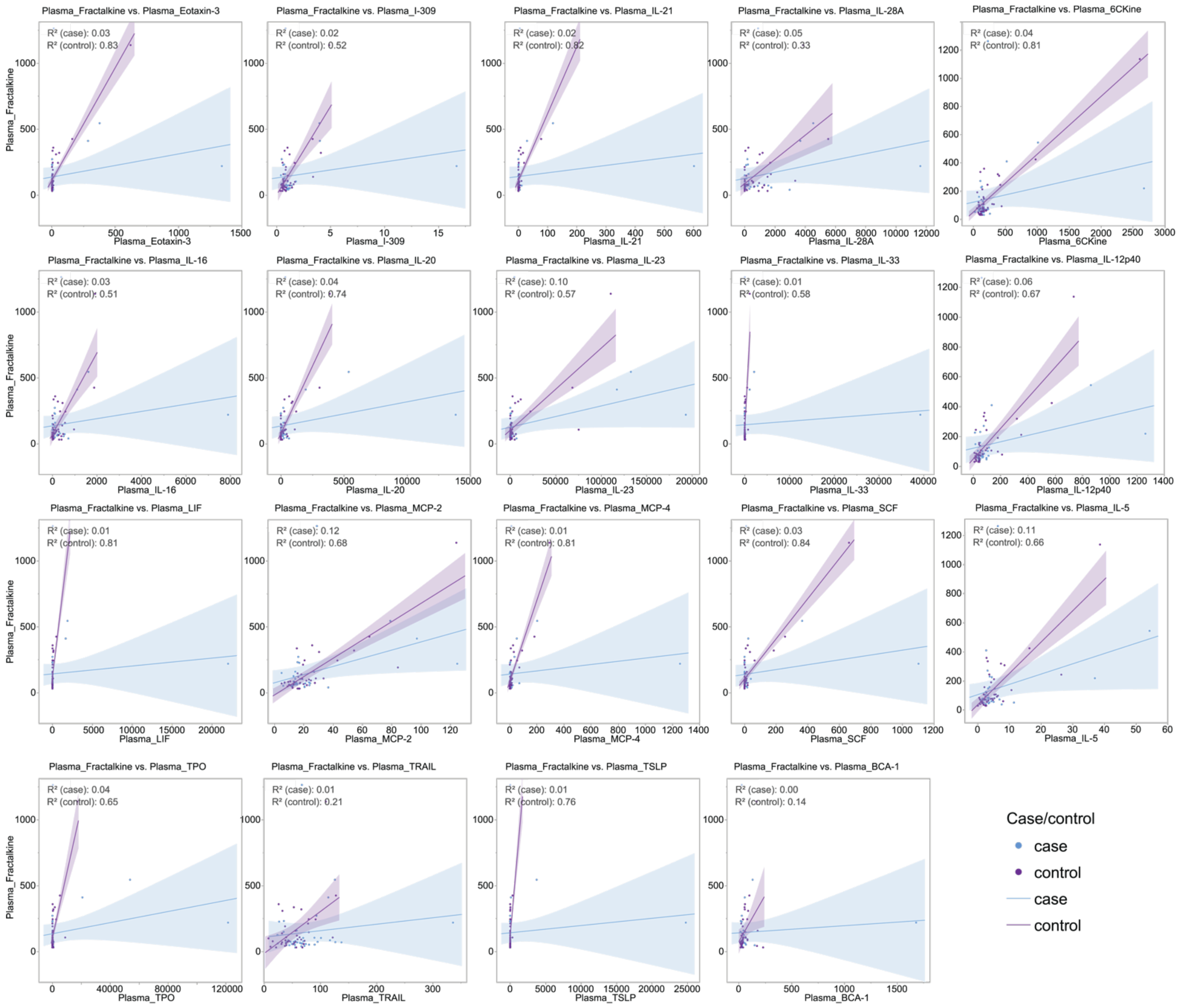

Supplementary Figure 4. Individual Pearson's correlation plots for measurements between plasma fractalkine and Eotaxin-3, I-309, IL-21, IL-28A, IL-16, IL-20, IL-23, IL-33, LIF, MCP-2, MCP-4, SCF, TPO, TRAIL, TSLP, BCA-1, 6Ckine, IL-12p40, and IL-15 from individuals with ME/CFS (blue) and healthy controls (purple). R^2 reported on each correlation plot. All values are depicted in pg/mL.

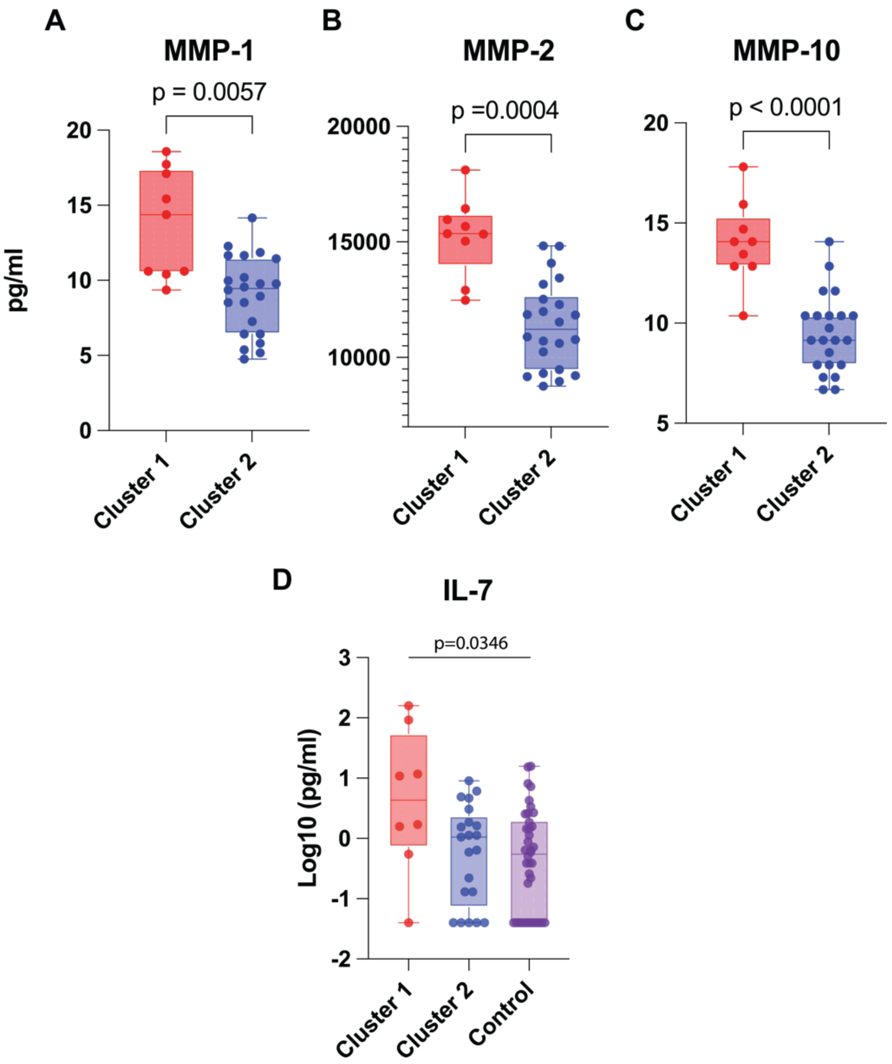

Supplementary Figure 5. A-C, Quantification of (A) MMP-1, (B) MMP-2, and (C) MMP-10 in CSF samples from cluster 1 (left, red) and cluster 2 (right, blue). Multiple comparison adjustment was performed using the False Discovery Rate method. **D,** Quantification of plasma IL-7 from cluster 1 (red), cluster 2 (blue), and healthy controls (purple). Multiple comparison adjustment was performed using the False Discovery Rate method. Significance was calculated by two sample t-testing accounting for variations in age, sex assigned at birth, and BMI between clusters through analysis of covariance (ANCOVA). The central lines indicate the group median, the top and bottom lines indicate the 75th and 25th percentiles, respectively.
